# Dislocation and oxygen-release driven delithiation in Li_2_MnO_3_

**DOI:** 10.1038/s41467-020-18285-z

**Published:** 2020-09-08

**Authors:** Kei Nakayama, Ryo Ishikawa, Shunsuke Kobayashi, Naoya Shibata, Yuichi Ikuhara

**Affiliations:** 1 Institute of Engineering Innovation, University of Tokyo, Bunkyo, Tokyo 113-8656 Japan; 2 PRESTO, Japan Science and Technology Agency, Kawaguchi, Saitama 332-0012 Japan; 3 Nanostructures Research Laboratory, Japan Fine Ceramics Center, Nagoya, 456-8587 Japan

**Keywords:** Batteries, Transmission electron microscopy

## Abstract

Lithium-excess layered cathode materials such as Li_2_MnO_3_ have attracted much attention owing to their high energy densities. It has been proposed that oxygen-release and cation-mixing might be induced by delithiation. However, it is still unclear as to how the delithiated-region grows. Here, by using atomic-resolution scanning transmission electron microscopy combined with electron energy-loss spectroscopy, we directly observe the atomic structures at the interface between pristine and delithiated regions in the partially delithiated Li_2_MnO_3_ single crystal. We elucidate that the delithiated regions have extensive amounts of irreversible defects such as oxygen-release and Mn/Li cation-mixing. At the interface, a partially cation disordered structure is formed, where Mn migration occurred only in the specific Mn/Li layers. Besides, a number of dislocations are formed at the interface to compensate the lattice mismatch between the pristine and delithiated regions. The observed oxygen-release and dislocations could govern the growth of delithiated-regions and performance degradation in Li_2_MnO_3_.

## Abstract (web summary)

The delithiation process in lithium excess cathode material of Li_2_MnO_3_ has remained unclear. Here, the authors report that delithiation is governed by the climb motion of dislocations with the aid of oxygen-release, determined directly by atomic-resolution STEM imaging and spectroscopy.

## Introduction

Li-ion secondary batteries are the most familiar and essential energy storages for consumer electronics, and plenty of efforts has been devoted to improving their capacities and cycle properties^1–4^. To achieve higher capacities, cathode materials with much higher Li contents have been explored. One of the potential candidates is Li-excess layered oxides of Li_2_MnO_3_ and the relatives of Li_(4−*x*)/3_Mn_(2−2*x*)/3_TM_*x*_O_2_ (TM = Ni, Co, etc.) because of their higher Li contents. This leads to the significantly higher theoretical capacities (up to 460 mAh g^−1^) than that of the current standard material of LiCoO_2_ (280 mAh g^−1^)^5–10^. In practice, however, the Li-excess oxides show severe degradations during charge/discharge cycles, which is significant even in the first or second cycles^11,12^. It is therefore important to understand in detail the delithiation process of Li_2_MnO_3_. Previous studies have proposed that Li_2_MnO_3_ may undergo two types of structural modifications during charge/discharge cycles: (i) oxygen release (the loss of oxygen in a sample), (ii) Mn migrations into Li sites that results in a Mn/Li disordered rocksalt-like structure, which have been extensively investigated by various methods^13–16^. To elucidate the relationship between such modifications and lattice defects, it has been investigated by scanning transmission electron microscopy (STEM)^13,14,17,18^. However, to our best knowledge, there is no report on detailed atomistic defect analysis because of a lack of highly delithiation-controlled specimen preparation such as using single crystals. Although such structural changes are often referred to as an underlying degradation mechanism, there is still the mystery of why the premade oxygen-deficient and/or cation-disordered specimens exhibit rather good battery performances^19–22^. This situation requires a more comprehensive insight beyond the viewpoint of the proposed structural modifications induced by delithiation processes. For example, because the oxygen release could lead to an increase in the lattice parameters, it is expected that the cycled cathode experiences large volume expansions. This would introduce a large non-uniform strain or lattice defects such as dislocations in the sample, which are very different from the case of the premade oxygen-deficient sample. It is therefore important to investigate a distribution of the strain fields and the presence of dislocations, which has recently been proposed by in situ Bragg coherent diffractive imaging technique^23^ and in situ TEM observation^24^. However, owing to the limitation of spatial resolution, the properties of strain field and dislocations introduced by charge/discharge process are still unclear.

Here, we show the direct determination of the interfacial atomic structure between pristine and delithiated regions in a partially delithiated Li_2_MnO_3_ single crystal by using atomic-resolution annular bright-field and dark-field (ABF/ADF) STEM combined with electron energy-loss spectroscopy (EELS). In the vicinity of the interface, we found a formation of partially cation mixed interfacial atomic structure with a thickness of 1–2 nm, and dislocations are also formed within the interface and in the delithiated region. We observed a certain amount of oxygen release near the surface of the delithiated regions, leading to the volume expansion. To compensate the lattice mismatch between the pristine and delithiated regions, we found the formation of dislocations at the interface. During delithiation, Li and O atoms should go through the interface, and therefore the climb motion of dislocations and oxygen-release process should govern the growth of the delithiated region. We note that the delithiation was performed by the chemical method using NO_2_BF_4_ oxidizer rather than an electrochemical reaction, which may damage the surface structure and also lead to the different structure evolution process. However, the present delithiation is rather slow chemical reaction (<10 nm thick with 16 h), and hence the surface damage could be sufficiently suppressed. Moreover, the observed oxygen release and cation mixing in Li_2_MnO_3_ have also been reported by the electrochemical reaction process^13–16^, which strongly supports the validity of the present study. We therefore conclude that the observed delithiation process with our chemical method could be well equivalent to the electrochemical reaction process.

## Results

### Delithiation and oxygen release in a Li_2_MnO_3_ single crystal

**Fig. 1:**
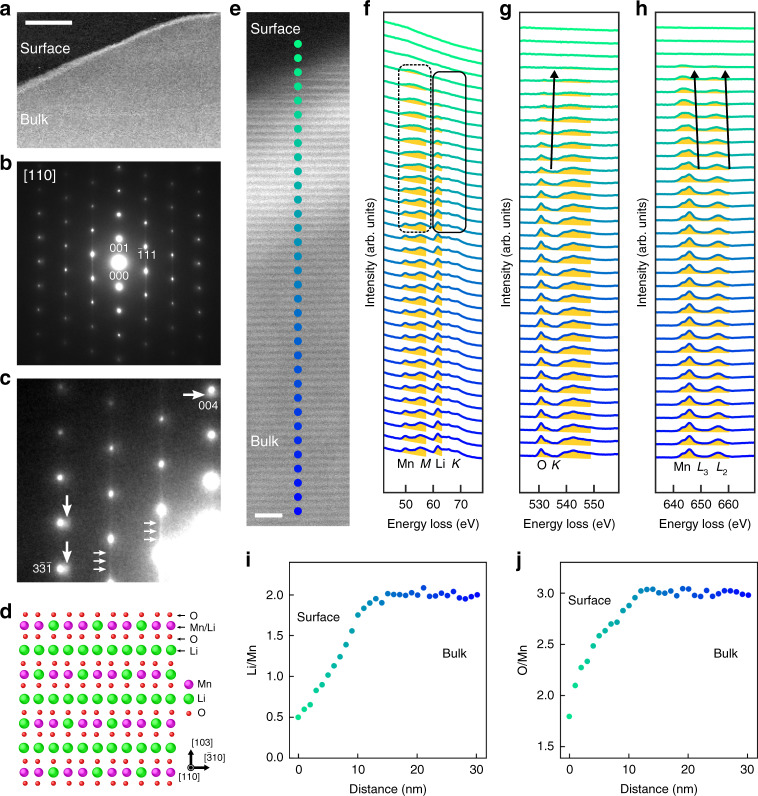
Determination of local chemical composition in delithiated Li_2_MnO_3_. **a** LAADF-STEM image and **b** corresponding electron diffraction pattern obtained from a partially delithiated Li_2_MnO_3_ single crystal. **c** Magnified electron diffraction pattern of **b**, where the contrast is intentionally enhanced. **d** The structure of Li_2_MnO_3_, viewed along the [110] direction (the notation is based on the monoclinic crystal system). **e** LAADF-STEM image obtained from the partially delithaited sample. **f**–**h** The EEL spectra obtained from the locations indicated by the circles in **e**: **f** Mn-*M*, Li-*K* edges, **g** O-*K* edge, **h** Mn-*L*_2,3_ edges, respectively. **i**, **j** The atomic ratios of Li/Mn and O/Mn calculated from the integrated intensities as a function of distance in **f**–**h**. The scale bars in **a** and **e** are 100 and 2 nm, respectively.

**Fig. 2:**
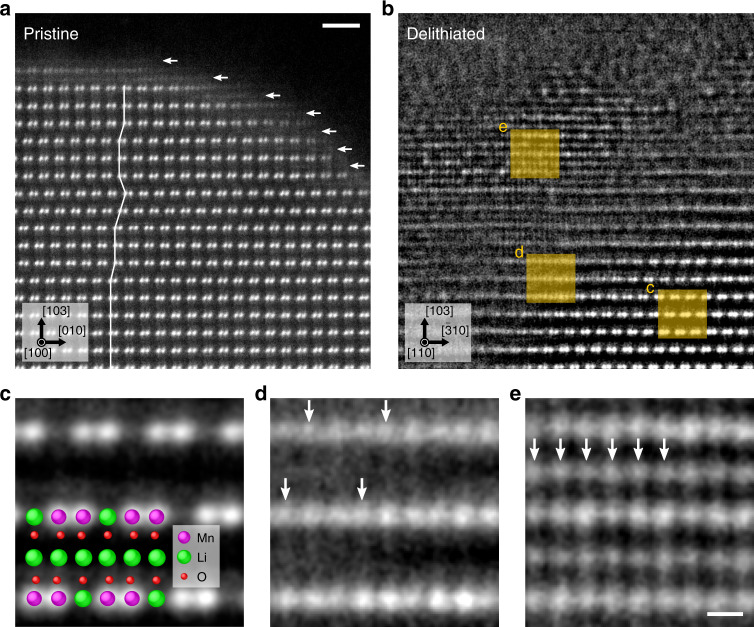
Atomic structural analysis in pristine and partially delithiated Li_2_MnO_3_ single crystals. HAADF-STEM images obtained from **a** pristine, **b** partially delithiated samples, respectively. The magnified HAADF-STEM images of orange squared regions: **c** pristine, **d** interface, and **e** delithiated regions, respectively. The scale bars in **a** and **e** are 1 nm and 0.2 nm, respectively.

Figure 1a, b shows a low-angle ADF (LAADF) STEM image and a corresponding selected area electron diffraction pattern obtained from the partially delithiated Li_2_MnO_3_, viewing along the [110] direction (monoclinic system). Figure 1c shows a magnified and intentionally contrast enhanced electron diffraction pattern of Fig. 1b. The corresponding structure model of Li_2_MnO_3_ is given in Fig. 1d (see also Supplementary Fig. 1), and the structure consists of the alternative stacks of Li, O, Mn/Li, and O layers along the [103] direction. Although all the sharp Bragg reflections in Fig. 1c are indexed by Li_2_MnO_3_, additional faint reflections are observed slightly inside the Bragg reflections of Li_2_MnO_3_, as marked by large white arrows in Fig. 1c. This suggests the delithiated phase has larger lattice spacings (or volume expansion) in both directions of [103] and $[\bar 310]$ than those in the bulk of Li_2_MnO_3_. Weak diffuse streaks are also observed along the [103] direction, as indicated by the small white arrows in Fig. 1c, and these streaks can be ascribed to stacking faults on the Mn/Li layers^25,26^. These stacking faults are also observed in the pristine sample (see Fig. 2a) and therefore the stacking faults may not be introduced by the delithiation process. It is noteworthy that the observed stacking faults are the simple translation of Mn/Li layers along the [010] direction, where the local environment (site symmetry) for Li atoms is basically the same as the original one. Moreover, the Li-ion diffusion path in this system is three dimensions and it is therefore such defects may not have a strong effect on the delithiation process. Figure 1e–h shows a LAADF-STEM image at a higher magnification and a set of EEL spectra obtained from the inside to the surface of the specimen as marked by circles in the LAADF-STEM image, where low losses of Li-*K* and Mn-*M* edges and core-losses of Mn-*L* and O-*K* edges were simultaneously recorded, respectively. In the LAADF-STEM image, the observed fringes correspond to the lattice spacing between adjacent Mn/Li layers. In Fig. 1f, compared with the low-loss Mn-*M* edge (49 eV), the peak intensity of the Li-*K* edge (60 eV) becomes smaller towards the surface, indicating that the Li concentration is reduced near the specimen edge. Although, at the low-loss Mn-*M* edge, two distinct peaks are observed in the pristine region, these peaks are merged into a single broaden peak near the surface as indicated by the dashed rounded square in Fig. 1f. This suggests that the Mn oxidation state is partially reduced from 4+ near the surface^13^. This is also confirmed by the downward chemical shift at the core-loss of Mn-*L* edge, as indicated by the arrows in Fig. 1h^13^. Normally, the charge variation introduced by a moderate delithiation in a reversible charge/discharge range is compensated by the oxidation of transition metal in the other system such as LiCoO_2_. However, in the present case, the oxidation state of Mn is rather reduced in the delithiated region and therefore, the formation of anion redox such as O_2_^*n*–^ (*n* ≤ 3) or oxygen release should be required for the charge neutrality in the system^16,27^. This situation is directly confirmed by the upward shift of O-*K* edge near the surface as indicated by the arrow in Fig. 1g^28^, suggesting that oxygen vacancies are introduced during delithiation, i.e., oxygen release. To estimate the local elemental concentrations, we evaluate the Li/Mn and O/Mn ratios by the integrated intensities of Li-*K*, Mn-*M*, O-*K,* and Mn-*L* edges, where the intensity integrations were performed through the yellow filled-regions in Fig. 1f–h. In the bulk region, the respective ratios of Li/Mn and O/Mn are known to be 2 and 3, which is used for the calibration to estimate the local elemental concentration from the integrated intensity. Assuming the linear relationship between the concentration and the integrated intensity, the resultant ratios were scaled and plotted as a function of distance in Fig. 1i, j, respectively. We note that the specimen thickness becomes thinner toward the surface, and we therefore used these atomic ratios rather than the integrated values to eliminate the thickness effect for the estimation of local elemental concentrations. It is clearly shown that the amounts of both Li and O atoms are reduced toward the surface, suggesting that the charge neutrality for the delithiation is mainly achieved by oxygen release. We note that the observed thickness of oxygen-release region (<10 nm) and the resultant fine structure change of O-*K* edge are well consistent with the previous reports via electrochemical delithiation^13,14^, suggesting that the present chemical delithiation process could be equivalent to that in electrochemical delithiation.

### Atomic structural analysis of the delithiated Li_2_MnO_3_

Figure 2a, b shows high-angle ADF (HAADF) STEM images obtained from pristine and partially delithiated Li_2_MnO_3_ single crystals, respectively. Owing to the Z-contrast nature in HAADF-STEM (Z is atomic number), the bright spots indicate the locations of heavy Mn atomic columns^29^. In the pristine sample, we observed the following two types of defects: (i) stacking faults^30,31^, and (ii) Mn/Li cation mixing in which a small amount of Mn atoms migrates into Li sites, as indicated by the line and arrows, respectively. These results suggest that the formation energy of the stacking faults may be considerably low, and the cation ordered bulk structure could be unstable at the surface. Although the cation mixing is inevitable at the surface region, the thickness is considerably thin of 1 nm. In Fig. 2b, although the pristine single crystal was oxidized by the considerably strong condition (16 h, see Methods), the size of the delithiated region was <10 nm, suggesting that the delithiation rate is considerably slow in this system. We found three-types of local atomic structures; a partially delithiated new structure is formed between the pristine and delithiated structures, as shown by the magnified HAADF-STEM images in Fig. 2c–e. The magnified images were obtained from the orange squares in Fig. 2b, where a sequential averaging method was employed to improve the signal-to-noise ratio of the images^32^. At the interface of Fig. 2d, Z-contrast intensities of all the atomic columns in the Mn/Li layer are basically uniform, suggesting that Mn atoms have migrated into the partially removed Li sites as indicated by the arrows, and partially cation disordered structure was newly formed. At the surface, as shown in Fig. 2e, Mn atoms are distributed into the remaining Li layers, and the Z-contrast intensities are close in almost all the cation atomic columns: the formation of Mn/Li fully disordered structure. Cation mixing should be related to the local Li concentration, and it is therefore the observed structure variations in Fig. 2c–e shows the process of the structural evolution induced by the delithiation. Namely, Mn migration (or mixing) occurs firstly in the Mn/Li layers at the interface and subsequently in the Li layers to be the delithiated region.

### Dislocations at the interface and in the delithiated region

**Fig. 3:**
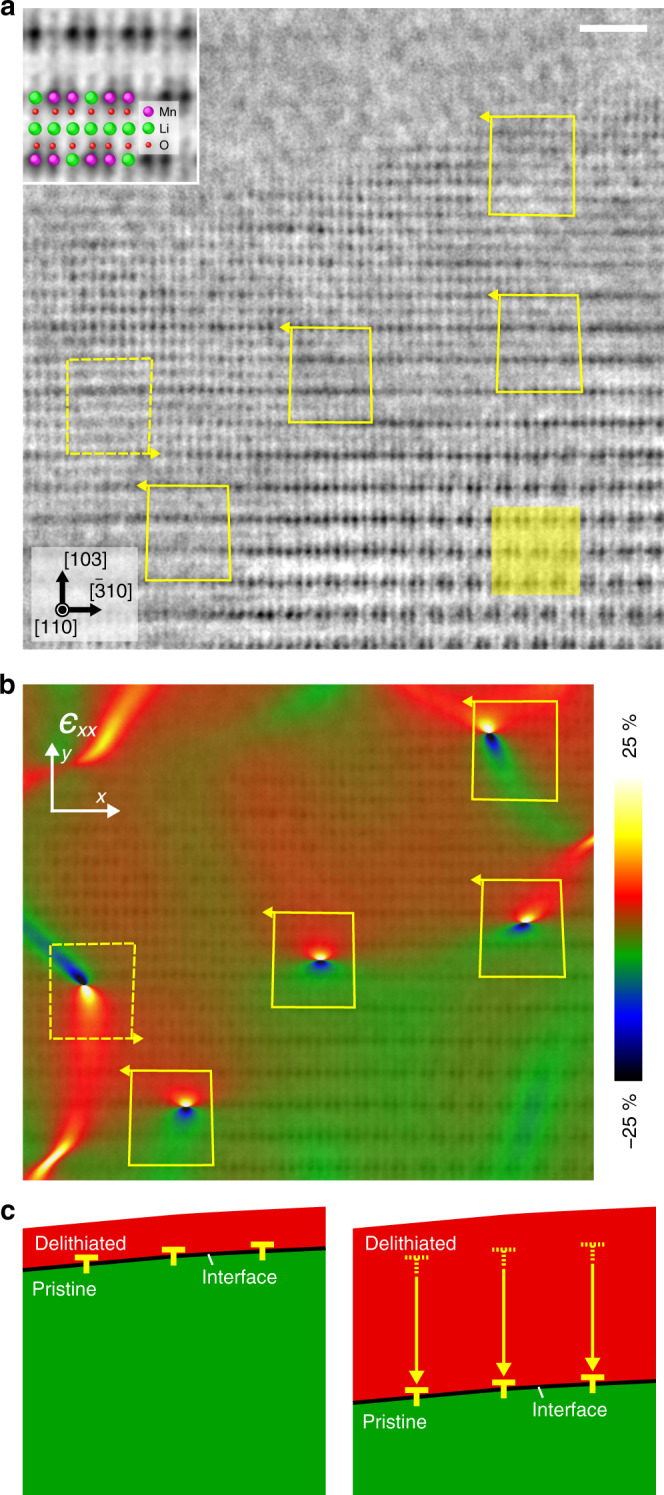
Atomic-resolution ABF-STEM imaging and GPA analysis in delithiated Li_2_MnO_3_. **a** ABF-STEM image simultaneously recorded with Fig. 2b. The inset is an enlarged image of the yellow squared region as a bulk. **b** The *ϵ*_*xx*_ strain map obtained from **a**. **c** A schematic view of delithiation process. The scale bar in **a** is 1 nm.

Figure 3a shows an ABF-STEM image^33^ simultaneously recorded with Fig. 2b, where the dark dotted contrasts correspond to the locations of both heavy and light atomic columns (see a magnified image in the inset). As noted in the electron diffraction analysis, the delithiated region shows the volume expansion, and dislocations could be formed at the interface to compensate the lattice mismatch between the pristine and delithiated regions. It is noteworthy that the main cohesive force of Li_2_MnO_3_ can be regarded as Coulomb attraction. Therefore, the oxygen release would induce the volume expansion because the magnitude of attractive force becomes weaker by the formation of oxygen vacancies, which is well consistent with the observed volume expansion in the premade oxygen-deficient Li_2_MnO_3−δ_^21,34^. On the close inspection of ABF-STEM image in Fig. 3a, we find three dislocations at the interface and two dislocations in the delithiated region. By drawing the Burgers circuits, the edge component (**b**_e_) of the dislocation is determined to be 1/12$[3\bar 10]$. Although we could not determine the screw component (**b**_s_) of the dislocation from the observation parallel to the [110] direction, the possible Burgers vectors are as follows: (i) **b** = 1/6[310] or 1/3$[0\bar 10]$ (**b**_s_ = ±1/4[110]) to be a pseudo-perfect dislocation, (ii) **b** = 1/3[100] or 1/6$[1\bar 10]$ (**b**_s_ = ±1/12[110], assuming face-centered cubic cation framework) to be a partial dislocation. We further performed geometric phase analysis (GPA) of the ABF-STEM image (see Methods), and it reveals that (i) the delithiated region (red-colored) has a larger volume than that in the bulk (green-colored) and (ii) considerable amounts of *ϵ*_*xx*_ strain fields are localized at the dislocations, as shown in Fig. 3b^35^. The dislocations at the interface have tensile strain on the side of the delithiated region, and therefore dislocations are introduced by the compensation of lattice expansions in the delithiated region. The average distance between these dislocations is roughly estimated to be 2.84 nm along the $[\bar 310]$ direction, which is well consistent with the calculated value of 3.5 nm from the average lattice mismatch by the electron diffraction in Fig. 1c (4.2%). We also found the other type of dislocation inside the delithiated region: the tensile strain field is located at the lower region, as marked by the dotted Burgers circuit in Fig. 3b. This result suggests that the volume expansion in the delithiated region could be non-uniform, which should be related to the non-uniform distribution of oxygen releases. The thickness of the oxygen-released region is ~10 nm that is significantly small amount, and the diffusion process for oxygen release could be considered as a result of a stochastic process (atomic scale), leading to the observed non-uniform volume expansion.

Here, we consider the growth process of the delithiated region, based on the motion of the observed dislocations. During delithiation, Li atoms are removed from the sample and cation (Li) vacancies are introduced. Simultaneously, oxygen vacancies via oxygen release are also introduced to compensate the charge neutrality in the system. Owing to the extraction of these Li and O atoms, delithiation starts from the surface and the dislocation would be introduced from the surface to compensate the lattice mismatch, where dislocations would be located at the interface between pristine and delithiated regions such as Fig. 3c. Next, Li and O atoms in the pristine region can diffuse into respective cation and anion vacancies in the delithiated region through the interface (from inside to the surface). During these migrations of Li and O atoms through the interface, both dislocations and the interface would be moved toward the opposite direction (from the surface to the inside) and the growth of delithiated region would be occurred. The motion of the dislocation should be climb motion, requiring diffusion of Li, O, and Mn atoms. However, as we have noted, many vacancies have already been introduced in the delithiated region and therefore both anion and cation may diffuse even at room temperature under the strong oxidized condition. Repeating these processes, delithiation will be promoted, accompanied by the climb motion of dislocations. However, these processes must be very slow and in fact the observed delithiated region is significantly small. To confirm the validity of our proposed delithiation growth mechanism, we also performed atomic-resolution observations for the lower degree of delithiation time of 8 h, as shown in Supplementary Note 1 and Supplementary Fig. 2. Even at the earlier stage of delithiation, we again observed the dislocations with the same Burgers vector at the interface, and we conclude that the growth of delithiation in Li_2_MnO_3_ system could be accompanied by the climb motion of dislocations.

## Discussion

In summary, to understand the delithiation process in Li-excess layered cathode materials, we have investigated the local atomic structure in the partially delithiated Li_2_MnO_3_ single crystal, by using atomic-resolution STEM imaging, GPA analysis and electronic structure analysis of EELS. We find that the formation of a partially cation mixed thin interfacial atomic structure between the pristine and the delithiated regions. In addition, dislocations are formed at the interface to compensate the lattice mismatch between the pristine and delithiated regions. As the formation of such dislocations is attributed to the local lattice expansion with oxygen release, the observed delithiation should be irreversible process. It is therefore the creation of dislocations would be contributed to the performance degradation issues such as energy capacity and durability. This result is very different from the case of Li_2_IrO_3_^36^, where the formation of peroxo-like species works as anion redox and oxygen release can be suppressed. Therefore, if we can form peroxo-like species in the Li_2_MnO_3_ system, the present irreversible defects could be reduced, leading to the improvement of the cycle performance. Another solution is a doping of transition metals such as Ni or Co in Li-excess system, where the oxygen release might be reduced because the charge neutrality for the delithiation can be achieved also by oxidation of the doped transition metals.

## Methods

### Specimen preparation

We used commercially available Li_2_MnO_3_ single crystals (Oxide Corp.), which are plate-shaped with a radius of 1.5 mm and a thickness of 10 μm (Supplementary Note 2 and Supplementary Figs. 3 and 4 show the sample characterization). A single crystal was mechanically cleaved by tweezers to expose fresh surfaces, and the obtained pieces were immersed in 20 ml acetonitrile solution of the NO_2_BF_4_ oxidizer with a concentration of 0.3 mol L^−1^ for 16 h in Ar atmosphere. After cleaning the samples three times by acetonitrile, the electron-transparent thin specimens for cross-sectional S/TEM observation were prepared by a focused ion beam (FIB) technique using NB5000 (Hitachi High-Technologies Co.) or Helios G4 (Thermo Fisher Scientific). Possible damage layers created by the Ga-ion beam in the FIB technique were removed by gentle Ar ion milling at 0.1–0.5 kV in PIPS II (Gatan, Inc.).

### Electron microscopy

Electron diffraction patterns and LAADF-STEM images were obtained by 2400FCS (JEOL Ltd.) operated at 120 kV. For LAADF-STEM, the probe-forming and the collection semiangles were 32 and 42–64 mrad, respectively. EEL spectra were obtained by a Tridiem-ERS EEL spectrometer (Gatan, Inc.), which is equipped with the 2400FCS microscope. These EEL spectra were recorded in STEM mode, using 0.2 eV per channel and an energy resolution of 0.8 eV (full-width at half-maximum of zero-loss peak). The convergence and collection semiangles were 32 and 42 mrad, respectively. Background signals were determined by power-low fitting. To estimate the Li/Mn ratios, the Li-*K* and Mn-*M* intensities were, respectively, integrated through ranges of 47.4–57.4 eV and 60.2–63.2 eV. To estimate the O/Mn ratios, the O-*K* and Mn-*L* intensities were, respectively, integrated through ranges of 528.8–548.8 eV and 640.2–660.2 eV.

The atomic structures around the interfaces between pristine and delithiated regions were investigated by ARM200CF (JEOL Ltd.) operated at 200 kV. The probe-forming aperture semiangle was 24 mrad, and ABF-STEM and HAADF-STEM images were recorded with the collection semiangles of 12–24 mrad and 90–200 mrad, respectively. Weiner filters and bandpass filters were applied to these images to reduce noise.

### Geometric phase analysis

The projected 2D strain tensor component of *ϵ*_*xx*_ was calculated by GPA, where we used the CrysTBox program (ver. 1.10)^37^. For the calculation of geometric phase, two basic vectors of **g**_1_ = 002 and **g**_2_ = $\bar 331$ in reciprocal space were selected in the Fourier transformed ABF image viewed along the [110] direction. The detailed theoretical background can be found in elsewhere^35^.

## Supplementary information

Supplementary Information

## Data Availability

The experimental data in this study are available from the corresponding author upon reasonable request.
