# Supplementary material for: Dislocation and oxygen-release driven delithiation in Li2MnO3
Source: Nat Commun. 2020 Sep 8;11:4452. doi: 10.1038/s41467-020-18285-z (PMC7479600; doi:10.1038/s41467-020-18285-z)
Supplement: Supplementary file 1 — Supplementary Information [file 41467_2020_18285_MOESM1_ESM.pdf]

Supplementary Information

Dislocation and oxygen-release driven delithiation in Li_2MnO_3

Nakayama et al.

Dislocation and oxygen-release driven delithiation in Li_2MnO_3

Kei Nakayama[1], Ryo Ishikawa[1,2], Shunsuke Kobayashi[3], Naoya Shibata[1,3], Yuichi Ikuhara[1,3,*]

[1]*Institute of Engineering Innovation, University of Tokyo, Bunkyo, Tokyo 113-8656, Japan*

[2]*Japan Science and Technology Agency, PRESTO, Kawaguchi, Saitama 332-0012, Japan*

[3]*Nanostructures Research Laboratory, Japan Fine Ceramics Center, Nagoya, 456-8587, Japan*

*Correspondence and requests for materials should be addressed to Y.I. (e-mail: ikuhara@sigma.t.u-tokyo.ac.jp)

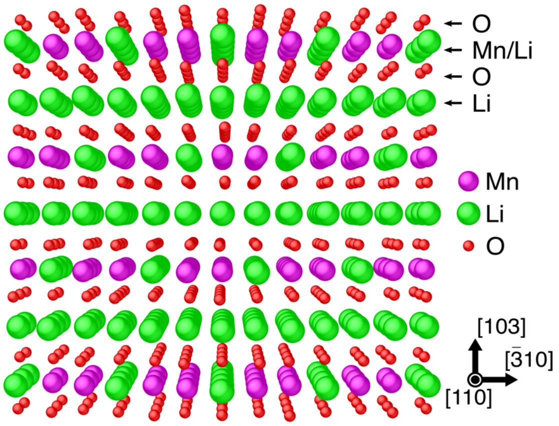

Supplementary Figure 1. A schematic diagram of the crystal structure of Li_2MnO_3 viewed along the [110] direction. The crystal structure is monoclinic symmetry and the space group is *C*2/*m* (ref. [1]). This structure can be regarded as an ordered rocksalt-type structure and is composed of Li, Mn/Li, and O layers with an ABC-type stacking sequence along the [103] direction.

Supplementary Note 1. To confirm the validity of the climb motion of dislocations during delithiation, we further observed the lower degree of delithiation via shorter immersing time of 8 h in NO_2BF_4 solution (originally 16 h in Fig. 3). Supplementary Figure 2 shows (a) atomic-resolution HAADF-STEM image and (b) the corresponding strain ϵ_{xx} map obtained from the delithiated/pristine interface. Compared to Fig. 3, the location of the interface is much closer to the specimen surface, suggesting that the present delithiation stage is earlier than Fig. 3b. In addition to the cation mixing at the delithiated regions, dislocations are observed at the interface, which is well consistent with the previously observation in Fig. 3. Therefore, it should be reasonable to conclude that the growth of delithiation could be accompanied with the climb motion of dislocations.

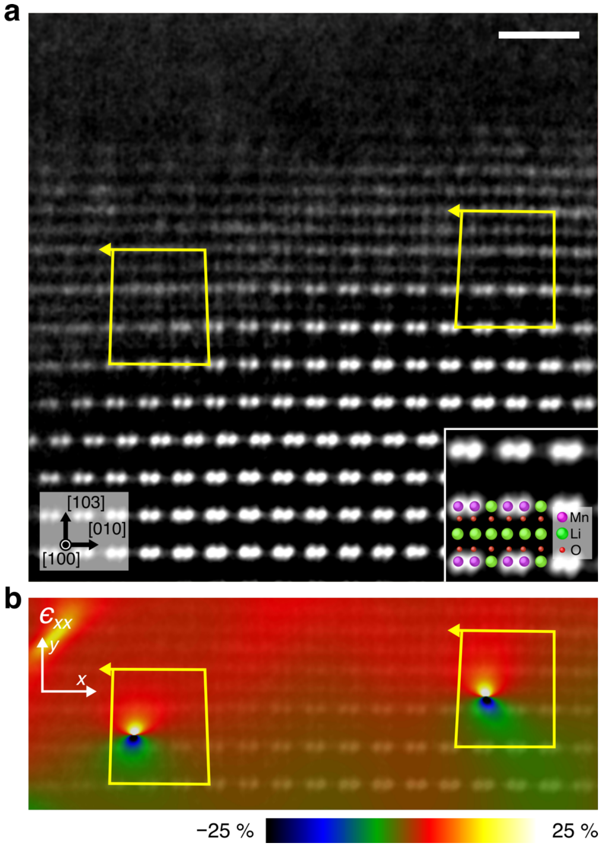

Supplementary Figure 2. a Atomic-resolution HAADF-STEM image of the interface between pristine and delithiated regions, where the single crystal was immersed in NO_2BF_4 solution at 8 h. The Burgers circuits are drawn by yellow lines. **b** The strain map of ϵ_{xx} derived from GPA analysis is overlaid on the corresponding HAADF-STEM image of **a**. The scale bar in **a** is 1 nm.

Supplementary Note 2. To characterize our pristine single crystals, we performed X-ray diffraction that is shown in Supplementary Fig. 3. The observed Bragg reflections are assigned to be $00l$ systematic reflections. This is because our single crystals are highly oriented along the $[103]$ direction (normal to the (001) plane), which could be found in the inset of optical image. For the further basic structural characterization, we also performed electron diffraction analysis in several crystal orientations and these results are well consistent with the previously reported Li_2MnO_3 structure. We note that no significant other phase or precipitations are not found in both diffraction analyses, and therefore our single crystals can be considered as the single phase.

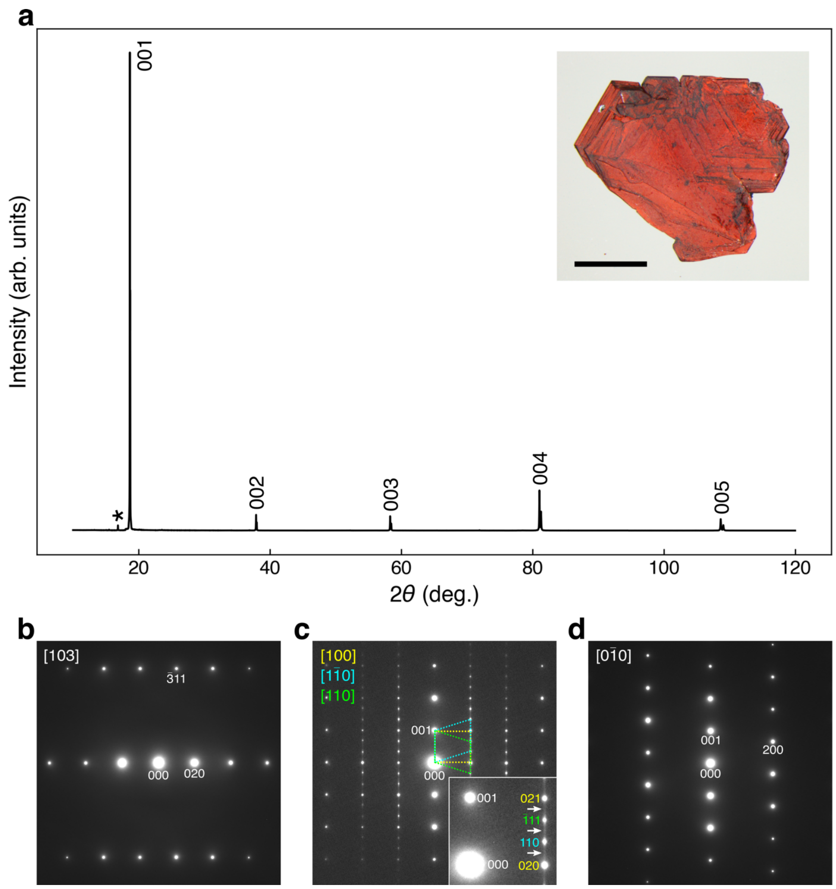

Supplementary Figure 3. a X-ray diffraction pattern obtained from the pristine single crystal (inset photograph). All the reflections are indexed by the monoclinic Li_2MnO_3 structure (ref. [1]). The asterisk indicates the existence of an unknow phase with a significantly small amount. **b** – **d** Selected area electron diffraction patterns obtained from the pristine single crystal viewed along the $[103]$, $[100]$ and $[0\bar{1}0]$, respectively, where all indices are the monoclinic Li_2MnO_3. The electron diffraction in **c** can be considered as the superimposed patterns of the $[100]$, $[1\bar{1}0]$, and $[110]$ (ref. [2]), and the diffuse streaks along the c-axis indicated by the arrows are attributed to stacking faults on the Mn/Li layers (refs. [3, 4]). The scale bar in inset of **a** is 1 mm.

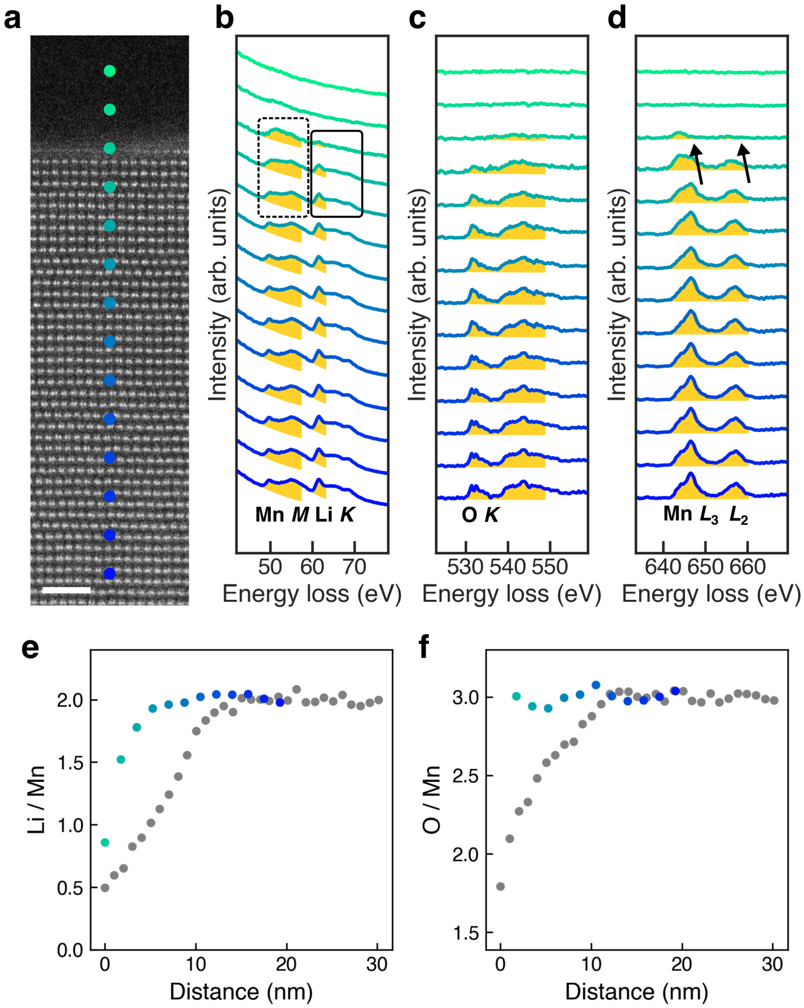

Supplementary Figure 4. a HAADF-STEM image obtained from the pristine sample. **b** − **d** EEL spectra obtained from the locations indicated by the circles in **a**: **b** Mn-*M*, Li-*K* edges, **c** O-*K* edge, **d** Mn-$L_{2,3}$ edges, respectively. Although **b** and **d** show spectral changes similar to those in Fig. 1**f** and **h**, respectively, they are found only in a few unit cells at the surface. **e, f** Calculated atomic ratios of Li/Mn and O/Mn from the integrated intensities in **b** − **d**, respectively. The grey circles indicate the values obtained from the delithiated sample (see Figs. 1**i**, **j** in main text). Li/Mn variation was observed only in a few unit cells at the surface and no significant oxygen release was detected. The scale bar in **a** is 2 nm.

Supplementary References

1. Strobel, P. & Lambert-Andron, B. Crystallographic and magnetic structure of Li_2MnO_3. *J. Solid State Chem.* **75**, 90-98 (1988).
2. Yu, H. et al. Direct Atomic-Resolution Observation of Two Phases in the $Li_{1.2}Mn_{0.567}Ni_{0.166}Co_{0.067}O_2$ Cathode Material for Lithium-Ion Batteries. *Angew. Chem. Int. Ed.* **125**, 6085-6089 (2013).
3. Boulineau, A., Croguennec, L., Delmas, C. & Weill, F. Reinvestigation of Li_2MnO_3 structure: Electron diffraction and high resolution TEM. *Chem. Mater.* **21**, 4216-4222 (2009).
4. Boulineau, A., Croguennec, L., Delmas, C. & Weill, F. Structure of Li_2MnO_3 with different degrees of defects. *Solid State Ion.* **180**, 1652-1659 (2010).
